# Retrospective Study of Bacteriological Patterns and Antimicrobial Resistance Profiles of Mastitis in the Banat Region of Romania

**DOI:** 10.3390/antibiotics15020198

**Published:** 2026-02-11

**Authors:** Caius Stoichescu, János Degi, Eugenia Dumitrescu, Florin Muselin, Diana Brezovan, Romeo Teodor Cristina

**Affiliations:** 1 Department of Pharmacy and Therapeutics, University of Life Science “King Michael I” from Timisoara, 300645 Timisoara, Romania; stoichescufarm@yahoo.com (C.S.); eugeniadumitrescu@usvt.ro (E.D.); romeocristina@usvt.ro (R.T.C.); 2 Department of Infectious Diseases and Preventive Medicine, University of Life Science “King Michael I” from Timisoara, 300645 Timisoara, Romania; 3 Department of Toxicology and Toxicoses, Plant Biology and Medicinal Plants, University of Life Science “King Michael I” from Timisoara, 300645 Timisoara, Romania; florinmuselin@usvt.ro; 4 Department of Histology and Embryology, University of Life Science “King Michael I” from Timisoara, 300645 Timisoara, Romania; dianabrezovan@usvt.ro

**Keywords:** bovine mastitis, multidrug-resistant bacteria, antimicrobial susceptibility, *Staphylococcus aureus*, dairy cattle health

## Abstract

Background: Bovine mastitis is a leading cause of economic loss in dairy farming and is increasingly complicated by antimicrobial resistance (AMR), posing challenges to treatment and public health. Objectives: This study aimed to investigate the prevalence, bacterial etiology, and AMR patterns of mastitis pathogens in dairy herds from the Banat region of Romania. Materials and Methods: A retrospective analysis was conducted on 420 dairy cows from five localities. Mastitis diagnosis involved clinical examination, indirect tests (California Mastitis Test (CMT), R-Mastitest), and bacteriological culture. Antimicrobial susceptibility was assessed using the VITEK^®^ 2 system. Results: Out of 420 cows, 120 (28.6%) were diagnosed with mastitis. The predominant pathogens were *Staphylococcus aureus* (33.3%) and *Streptococcus agalactiae* (22.5%). Most infections were monomicrobial (70%) and affected a single under quarter (77.5%). Beta-lactam resistance was widespread among both Gram-positive and Gram-negative isolates, particularly against penicillin and ampicillin. Multidrug-resistant (MDR) strains were identified in 33.3% of all isolates, with 100% of Gram-negative isolates exhibiting MDR profiles. Conclusions: The high prevalence of *S. aureus* and *S. agalactiae*, along with widespread beta-lactam resistance and frequent MDR phenotypes, highlights the urgent need for routine AMR surveillance and targeted antimicrobial therapy in bovine mastitis control programs.

## 1. Introduction

Mastitis remains a major economic and welfare challenge in the global dairy industry, leading to reduced milk yield, inferior milk quality, increased veterinary costs, and early culling [1,2,3]. Globally, the prevalence of subclinical mastitis ranges from 25% to 50%, while clinical mastitis affects approximately 5–15% of lactating cows [1,2]. The economic loss per mastitis case has been estimated between USD $110 and $285, depending on severity and milk price [3,4]. Beyond its direct economic burden, mastitis is a key driver of antimicrobial use in livestock, which contributes to the global spread of AMR. Beyond its direct impact on animal health, mastitis poses significant public health concerns due to the complex interplay between host susceptibility, pathogen virulence, and environmental factors [1,5,6]. This triad underlies both the occurrence and recurrence of clinical and subclinical cases in dairy herds [7,8,9]. Recent reports indicate that over 70% of *S. aureus* mastitis isolates show resistance to penicillin and ampicillin, with MDR increasingly reported [8]. *S. agalactiae*, a contagious pathogen, also exhibits rising resistance, especially to tetracyclines and macrolides [9]. The detection of MDR phenotypes in both Gram-positive and Gram-negative mastitis pathogens poses serious challenges to effective therapeutic management.

Despite the routine use of antimicrobials in treatment, current, region-specific data from Romania on resistance patterns in mastitis pathogens, especially from small to medium-scale farms, remain limited. This data gap hinders effective antimicrobial stewardship and impairs our understanding of resistance transmission at the animal–human–environment interface.

Milk, as a complex biological fluid, provides a highly favorable medium for the survival and proliferation of numerous microbial species, some of which may have zoonotic potential. Even organoleptically altered milk from a single mastitic cow is sufficient to compromise the safety of bulk milk collected from entire herds, posing significant biosecurity risks [10,11]. The bacterial agents isolated from milk samples from cows with mastitis typically originate either from systemic circulation or directly from local inflammatory lesions within the under tissue [11,12,13,14]. This microbial contamination, if undetected, may persist throughout the production and distribution chain of dairy products, undermining both consumer safety and regulatory compliance. The emergence of mastitis pathogens exhibiting AMR extends beyond animal health, posing potential risks to public health through the food chain and the environment. This highlights the need to examine bovine mastitis within a One Health framework, particularly in regions where surveillance data are limited.

Within the One Health framework, the European Union has implemented coordinated surveillance and management strategies to address antimicrobial resistance across the human–animal–environment interface. Large-scale initiatives such as the Innovative Network for the Management of Antibiotic Resistance in Livestock Farming [15] and the DISARM project [16] aim to harmonize monitoring systems, promote prudent antimicrobial use, and develop sustainable mitigation strategies in livestock production. These programs emphasize the importance of region-specific epidemiological data to inform evidence-based antimicrobial stewardship policies.

Somatic cell count (SCC), composed primarily of leukocytes and exfoliated epithelial cells, is a well-established marker of mammary gland health and is influenced by multiple physiological and pathological factors, including lactation stage, immune status, and the secretory epithelium [17,18,19,20]. While these cellular components are naturally present in normal milk, their relative proportions change significantly in the context of intramammary infections. The increase in leukocyte infiltration reflects the innate immune response to pathogen invasion and serves as an early warning indicator for subclinical mastitis [21,22,23,24,25].

From an epidemiological standpoint, dairy cows are continuously exposed to environmental and opportunistic pathogens in their housing systems, making mastitis surveillance and control a permanent challenge [26,27]. Regional differences in pathogen prevalence, antimicrobial resistance profiles, and farm level hygiene practices underscore the need for context specific data to inform control strategies [28].

Despite the high burden of mastitis in Eastern European dairy systems, data on bacterial etiology and AMR patterns in Romania remain limited and fragmented. Most available studies rely on conventional bacteriology and are often confined to large-scale farms, with scarce data from small or medium-sized operations typical of western Romania. Specifically, in the Banat region—an area with both industrial and traditional dairy farms—there is a critical lack of up-to-date surveillance on mastitis pathogens and their resistance profiles. Moreover, the use of standardized automated systems such as VITEK^®^ 2 for AMR testing remains underreported in Romanian veterinary research. Given the increasing threat of AMR, the lack of regional data, and the limited adoption of automated diagnostics in Romanian veterinary microbiology, there is an urgent need to characterize the current spectrum of mastitis pathogens and their resistance traits. The present study aims to address this gap by providing a retrospective epidemiological overview of clinical and subclinical mastitis in dairy herds from five localities in the Banat region, with emphasis on species distribution, AMR patterns, and the prevalence of MDR isolates. These findings are expected to support evidence-based treatment protocols and contribute to national antimicrobial stewardship efforts within the One Health framework.

Therefore, the aim of the present study was to provide a retrospective epidemiological assessment of clinical and subclinical bovine mastitis in dairy herds from the Banat region of Romania, with particular emphasis on pathogen distribution, antimicrobial resistance patterns, and the prevalence of MDR isolates, in alignment with One Health surveillance priorities.

## 2. Results

Following the execution of indirect diagnostic tests (R-Mastitest and/or Mast-O-Test), the examined milk samples were classified as:

- 60 milk samples collected from cows with subclinical mastitis.
- 60 milk samples collected from cows with clinical mastitis.

Depending on the bacteriological criterion, the 120 mammary milk samples were divided into three categories of samples:

- 84 monomicrobial mastitis (70% of cases).
- 26 polymicrobial mastitis (21.66% of cases).
- 10 sterile mastitis (8.33% of cases).

The instantaneous prevalence index for bacterial mastitis in the investigated cows was 0.28, respectively 28.6% of the average herd of cows developed mastitis during the observation period.

Of the 420 cows investigated during the study period, 120 were classified as having either subclinical or clinical mastitis, corresponding to an overall prevalence of 28.6%.

In the present study, SCC values in subclinical mastitis cases ranged from 312,000 to 1,084,000 cells/mL, whereas clinical mastitis cases exhibited SCC values between 1,265,000 and 6,940,000 cells/mL. The higher SCC values observed in clinical cases reflect the intensified inflammatory response associated with over-udder pathology.

Bacteriological analysis of milk samples revealed that *S. aureus* was the most prevalent pathogen in monomicrobial mastitis, being isolated in 40 cases (47.61%). Other monomicrobial etiologies included *S. agalactiae*, *Streptococcus dysgalactiae*, *Streptococcus uberis*, *Streptococcus pyogenes*, *Corynebacterium bovis*, *Escherichia coli*, *Klebsiella pneumoniae*, and *Morganella morganii*, collectively accounting for 52.39% of the cases. Additionally, 26 samples (21.66%) yielded polymicrobial infection. In the 10 culture-negative mastitis cases, SCC values ranged from 358,000 to 1,420,000 cells/mL, with a mean value of approximately 820,000 cells/mL. Despite the absence of bacterial growth on routine culture, these elevated SCC values indicate an ongoing inflammatory response at the mammary gland level (Table 1, Figures S2 and S3—Supplementary File), potentially due to prior antibiotic treatment or suboptimal sampling conditions.

Milk appearance was classified into three predefined organoleptic categories:

(1) Normal (51 samples): matte white, normal consistency, without visible clots or flakes, despite positive diagnostic test results;
(2) Serous or clotted (43 samples): altered (white to yellow), with watery/serous or increased viscosity, frequently containing visible flakes or clots;
(3) Purulent (26 samples): grayish-yellow, reddish, or greenish secretion, typically viscous, with sediment formation and a supernatant of variable clarity.

Interestingly, no consistent correlation was found between the degree of physicochemical alteration in milk (color, viscosity, pH, conductivity, etc.) and the type of causative microorganism. For example, 75% of *S. aureus* infections are presented with apparently normal milk. Similarly, 23.07% of samples infected with *Streptococcus* spp. or polymicrobial infection showed no overt changes in milk appearance.

Acute cases of mastitis caused by *S. dysgalactiae* exhibited a distinctive milk phenotype watery, nearly transparent secretion. This consistent observation suggests a potentially specific organoleptic signature associated with *S. dysgalactiae*, which may aid in the preliminary clinical differentiation of mastitis etiology under field conditions. Moreover, infections with *S. agalactiae* were frequently persistent, aligning with previous literature that associates this species with chronic intramammary infections.

Based on the number of affected quarters, the 120 cows diagnosed with mastitis were stratified into four distinct categories: 93 cows (77.5%) exhibited infection in a single mammary quarter, 21 cows (17.5%) in two quarters, 5 cows (4.2%) in three quarters, and only one cow (0.8%) had all four quarters simultaneously affected (Table 1).

The results of this study indicate that, in most cases (77.5%), mastitis affected only a single mammary quarter. However, there were notable instances where two, three, or all four quarters were simultaneously affected, albeit to varying degrees of severity. Supporting this assumption, all infected quarters within the same animal were consistently found to harbor the same etiological agent (Table 1), suggesting local dissemination rather than multiple independent infections.

No clear correlation was identified between the type of pathogen and the number of infected quarters, with one exception: *S. aureus* infections were mostly localized, with only 5% of affected cows showing involvement of more than one quarter. In contrast, infections caused by *Streptococcus* spp. involved multiple quarters in 30.77% of cases, and polymicrobial mastitis was associated with bilateral involvement in 38.5% of animals.

Reduced milk yield, compromised product quality, and downgraded milk composition translate into tangible financial losses. Additionally, treatment costs including antibiotics and veterinary labor contribute to the overall economic burden of the disease.

When analyzed according to the lactation stage, the distribution of mastitis cases revealed the following pattern: 17 cases (13.6%) were diagnosed during the early lactation phase (first 1–2 months), 20 cases (16%) during mid-lactation (plateau phase), and 83 cases (66.4%) during the late lactation phase (final 1–2 months) (Table 1).

Antimicrobial susceptibility testing performed using the automated VITEK^®^ 2 system revealed distinct resistance profiles among the bacterial isolates involved in mastitis cases, reflecting both pathogen-specific patterns and the impact of antimicrobial exposure at herd level. For Gram-positive bacteria, testing was conducted using AST-GP cards, while Gram-negative isolates were evaluated using AST-GN cards, providing quantitative minimum inhibitory concentration values for each antimicrobial agent tested (Tables S1 and S2—Supplementary File).

Most *Staphylococcus aureus* isolates displayed elevated MIC values for penicillin and aminopenicillins, with inhibitory concentrations frequently exceeding the susceptibility thresholds established by Clinical and Laboratory Standards Institute [29]. Estimated MIC values for penicillin were generally above 1–2 µg/mL, indicating widespread beta-lactamase activity among the isolates. A subset of strains also exhibited reduced susceptibility to tetracycline and erythromycin, with MIC values typically ranging between 4 and 16 µg/mL, consistent with acquired resistance mediated by efflux pumps and ribosomal protection mechanisms. In contrast, susceptibility to oxacillin and first-generation cephalosporins remained preserved in most isolates, with MIC values for oxacillin typically below 0.5 µg/mL, excluding phenotypic methicillin resistance. Aminoglycosides such as gentamicin are retained high in vitro activity, with MICs commonly reported below 1 µg/mL. Fluoroquinolones also showed good efficacy, with enrofloxacin MIC values generally ranging between 0.25 and 1 µg/mL.

The streptococcal isolates were characterized by a favorable susceptibility profile toward beta-lactam antibiotics. Penicillin MIC values were consistently low, typically below 0.12 µg/mL, supporting their continued role as first-line therapy in streptococcal mastitis. However, a proportion of isolates demonstrated elevated MIC values for tetracycline, in the range of 8–16 µg/mL, and reduced susceptibility to macrolides, suggesting long-term selective pressure associated with these antimicrobial classes. Resistance to potentiated sulphonamides was occasionally observed, with MIC values exceeding 4 µg/mL for trimethoprim, although sensitivity to aminoglycosides remained largely preserved.

Gram-negative isolates such as *E. coli* and *K. pneumoniae* showed a heterogeneous resistance pattern. High MIC values for amoxicillin and ampicillin, frequently above 8–16 µg/mL, indicated constitutive or acquired beta-lactam resistance. Resistance to tetracycline was also common, with MICs often exceeding 8 µg/mL. A reduction in susceptibility to trimethoprim-sulfamethoxazole was observed in several isolates, while fluoroquinolones exhibited variable activity, with MIC values ranging from ≤0.25 to >2 µg/mL depending on the strain. Third-generation cephalosporins such as ceftiofur generally maintained activity against most isolates, with MIC values predominantly below 2 µg/mL, suggesting that extended-spectrum beta-lactamase production was not widespread in the studied material.

In polymicrobial mastitis cases, susceptibility profiles were frequently discordant between co-isolated species, often revealing at least one component with multi-resistant characteristics. In several samples, one isolate displayed elevated MIC values across at least three antimicrobial classes, fulfilling phenotypic criteria for multidrug resistance. This observation underscores the therapeutic challenge posed by mixed infections, where empirical therapy based on predictable susceptibility patterns may prove insufficient.

**Figure 1:**
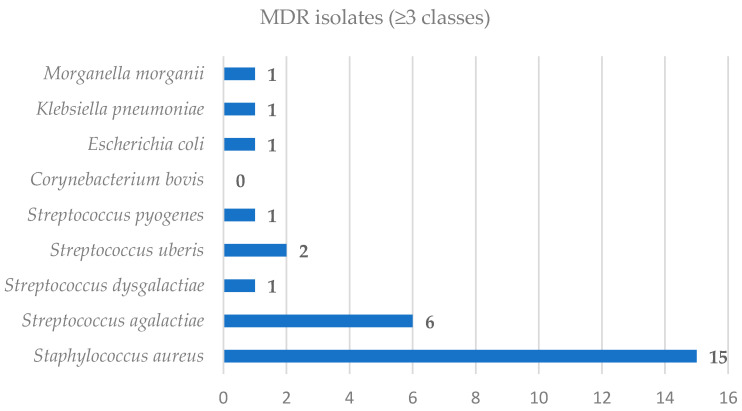
Distribution of MDR isolates among mastitis pathogens.

AMR is a growing concern in the management of bovine mastitis, with direct implications for treatment efficacy and herd health. Understanding the distribution and resistance profiles of mastitis-causing pathogens is essential for guiding therapeutic decisions and implementing effective control measures [4,5,8,24]. MDR, defined as resistance to three or more antimicrobial classes, was observed across several bacterial species. Table 2 and Figure 1 summarize the number and percentage of MDR isolates for each pathogen identified.

*S. aureus* showed the highest MDR frequency among Gram-positive isolates (37.5%), followed by *S. dysgalactiae*, *S. uberis*, and *S. pyogenes* (each 33.3%). *S. agalactiae* also demonstrated notable MDR prevalence (22.2%). Among Gram-negative bacteria, all isolates of *E. coli*, *K. pneumoniae*, and *M. morganii* (*n* = 1 each) were multidrug resistant, indicating 100% MDR rates.

A total of 84 bacterial isolates were analyzed, of which 28 (33.3%) were classified as MDR. Detailed species-level distribution is presented in Table S3. Table S4—Supplementary File provides a quantitative assessment of the proportion of MDR isolates for each bacterial species, along with 95% confidence intervals (CI) calculated using the Clopper–Pearson method, and statistical comparisons (*p*-values) between species.

A visual representation of these proportions (Figure S4—Supplementary File) highlights numerical differences in MDR prevalence across species; however, for most bacterial groups with low isolate counts, the broad confidence intervals reflect significant statistical uncertainty. Only *S. aureus*, and to a lesser extent *S. agalactiae*, offer sufficiently narrow confidence intervals to support meaningful epidemiological interpretation. These findings emphasize the need for larger datasets to enable more robust interspecies comparisons.

The diversity in the number and type of antibiotic classes tested between Gram-positive and Gram-negative species should be considered when comparing MDR rates across taxa.

The antimicrobial resistance patterns of the mastitis-associated bacterial isolates are summarized in Table 2 and illustrated in Figure 1.

The distribution of mastitis cases according to lactation phase and etiological agent is presented in Table 3. The data reflects how pathogen prevalence may vary throughout the lactation cycle, providing insights into infection dynamics and susceptibility periods.

Table 3 summarizes the number of mastitis cases associated with each identified pathogen during three lactation stages: early (30–60 days), mid (60–245 days), and late (245–305 days). Most cases (69%) occurred in the late lactation phase, with *S. aureus* and *Streptococcus* spp. being the most frequently isolated pathogens across all stages.

## 3. Discussion

According to current literature, in dairy operations where mastitis control and prevention programs are not systematically implemented, the incidence of both clinical and subclinical mastitis remains comparable to that reported 30–40 years ago. Under such conditions, up to 25% of cows may be affected by some form of mastitis [30]. In our study, the average morbidity rate was 28.6%, indicating that approximately one in four cows presented with either subclinical or clinical mastitis, a prevalence consistent with historical patterns in poorly controlled herds.

While some pathogens isolated from normal-looking milk may later lead to overt clinical mastitis, others may persist in a latent state or even self-resolve without treatment. Nonetheless, the mammary immune system does not remain passive. Subclinical mastitis is often accompanied by an increase in SCC, primarily due to elevated numbers of phagocytic cells, and is reflected by changes in pH, enzymatic activity, electrical conductivity, and other milk quality indicators.

Although mastitis is rarely fatal, five cases of cow culling due to unresponsive infections were recorded during the study. These animals exhibited extensive mammary pathology, including nodular lesions, advanced quarter atrophy, and infection involving three or four quarters. All five cases demonstrated continued antimicrobial resistance despite treatment, leading to removal from the productive herd. While the animals were not lost due to acute systemic failure, their premature culling rendered them economically unviable. Based on this, a functional “lethality index” of 5% can be estimated, highlighting the potential for mastitis to cause irreversible economic loss even without direct mortality.

Although intramammary infections can occur at any point during the lactation cycle, our data together with previous reports suggest that many infections are sub clinically established during the mammary resting period, particularly during active involution and colostrogenesis, and may only become clinically manifest at calving or later [31,32].

Interestingly, in contrast to these well-documented trends, our study revealed that the highest frequency of mastitis cases (69%) occurred in late lactation (Table 3). This deviates from the classical pattern reported in the literature, where early lactation is typically associated with peak incidence [18,33,34,35]. This atypical distribution may be explained by local risk factors, particularly deficiencies in milking hygiene and delayed or absent intralactational treatment, leading to cumulative infection pressure and pathogen load toward the end of the lactation cycle.

The discrepancy between our observations and those reported in the literature regarding the typical timing of mastitis onset usually during early lactation may be primarily attributed to significant deficiencies in the mechanical milking process observed in all investigated herds. Improper functioning or inadequate maintenance of milking equipment likely resulted in chronic teat-end trauma and increased exposure to pathogens, progressively predisposing cows to intramammary infections as lactation advanced.

Because of this persistent risk factor, the cumulative number of mastitis cases increased over time, frequently peaking toward the end of the lactation period. A secondary but complementary explanation lies in the absence of systematic treatment for mastitis during lactation. This therapeutic gap likely contributed to elevated infectious pressure and bacterial load within the herd, facilitating new infections and the persistence of subclinical cases. Together, these factors provide a plausible rationale for the observed shift in the peak incidence of mastitis from early to late lactation in our study population.

The distribution of affected quarters may reflect intra-mammary transmission dynamics within individual animals. Although most cases involve a single quarter, the presence of multi-quarter infections suggests that pathogens may spread locally over time, particularly in the absence of early intervention. Intra-mammary dissemination has been previously described, especially for contagious pathogens such as *S. aureus* and *S. agalactiae*, which can spread during milking through contaminated equipment or improper hygiene practices [5,8,36].

The findings of the present study underscore several important epidemiological and clinical aspects of bovine mastitis. The frequent occurrence of subclinical cases in the absence of visible milk alterations confirms that mastitis may persist undetected without systematic screening, emphasizing the importance of routine diagnostic monitoring. The predominance of single-quarter involvement suggests that most infections remain localized; however, the presence of multi-quarter cases supports the possibility of intra-mammary transmission, whereby infection may spread from one quarter to adjacent ones over time. Differences in the extent of quarter involvement between pathogens may reflect variations in pathogenicity, tissue tropism, and transmission dynamics. The tendency for mastitis cases to accumulate toward late lactation further suggests progressive infection pressure within herds, potentially influenced by management factors. From an economic standpoint, partial or complete functional impairment of affected quarters contributes significantly to production losses. Additionally, the considerable proportion of multidrug-resistant isolates detected in this study reinforces the growing concern regarding antimicrobial resistance among mastitis pathogens and its implications for effective herd-level control strategies.

In addition to identifying the etiological agents responsible for bovine mastitis, the antimicrobial susceptibility profiles of the isolates represent a critical component for effective clinical intervention and the development of rational herd-level antimicrobial stewardship strategies [2,3,5,37]. Addressing one of the key reviewer comments, our study employed the VITEK^®^ 2 automated system for standardized susceptibility testing of both Gram-positive and Gram-negative isolates, thereby ensuring reproducibility and comparability with other international reports [9,27,33,37].

The predominance of *S. aureus* in our findings reflects not only its wide distribution in dairy herds but also its remarkable capacity for chronic intramammary persistence [5,6,8,21,26,37]. An important clinical observation emerging from this study is the marked difference in the degree of organoleptic alteration of milk between mastitis caused by *Streptococcus* spp. and *Staphylococcus* spp. In 76.93% of streptococcal mastitis cases, visible changes in milk appearance were recorded, including altered consistency, and the presence of abnormal secretions. These changes were notably more pronounced compared to staphylococcal mastitis, which often remains subclinical or presents with fewer sensory modifications [5,8,21,26].

Several factors contribute to this dominance: its ability to form biofilms on mammary epithelium and milking equipment surfaces, which protects bacterial colonies from host immune responses and antimicrobial agents [6,38,39]; its capacity for intracellular survival within mammary epithelial cells, allowing it to evade phagocytosis and sustain latent infections [6,17,37]; and its efficient transmission via contaminated milking equipment, facilitating herd-level dissemination [4,6,40,41]. These features explain not only the high recurrence of *S. aureus*-associated mastitis, but also the accumulation of resistance traits that complicate therapeutic management.

All streptococcal isolates demonstrated MIC values within the CLSI-defined susceptibility breakpoints for both macrolides and tetracyclines. However, the presence of resistance determinants in a subset of isolates suggests potential for emerging resistance. Clinicians should therefore consider local resistance trends and the possibility of future shifts when selecting empirical therapy.

Despite the limited statistical power observed in certain species groups due to the relatively small number of isolates, the results indicate a considerable burden of MDR among both Gram-positive and Gram-negative mastitis pathogens. As detailed in Table S5 (Supplementary File), MDR status appears to be influenced not only by species prevalence but also by the extent of antimicrobial exposure within the herd. *S. aureus* emerged as a major contributor to the overall MDR burden, combining high prevalence with substantial resistance levels. In contrast, although Gram-negative species were isolated infrequently, they exhibited a 100% MDR rate, reflecting broad-spectrum resistance patterns that may significantly complicate therapeutic decision-making and increase the potential clinical impact of these infections and highlights the potential role of bovine mastitis as an interface in the One Health AMR ecology. Environmental reservoirs such as manure, bedding, and slurry may act as sources or sinks for MDR bacteria, facilitating persistence and spreading via horizontal gene transfer mechanisms. These findings underscore the need for integrated surveillance and mitigation strategies that address AMR beyond the clinical setting [1,3,10,35].

This resistance pattern may reflect regional antimicrobial usage practices, where penicillin-based intramammary preparations remain the most used therapeutic agents, both during lactation and as part of blanket dry cow therapy protocols [37,41]. In the absence of routine antibiogram testing, the empirical and repeated use of these agents can apply prolonged selective pressure, favoring the survival of β-lactam–resistant *S. aureus* strains. Additionally, limited regulatory oversight and uneven implementation of antimicrobial stewardship principles across small- and medium-scale Romanian dairy farms may exacerbate this trend.

Nonetheless, preserved susceptibility to third-generation cephalosporins and, partially, to fluoroquinolones was encouraging, indicating that these molecules may retain therapeutic value when applied judiciously and under veterinary supervision [2,26].

Our findings contribute to the growing body of evidence linking antimicrobial resistance in dairy pathogens to broader One Health concerns. The detection of resistant strains in milk underscores not only challenges in therapeutic management but also potential pathways for resistance gene transmission to humans via direct contact, food consumption, or environmental exposure.

The SCC ranges recorded using on-farm Direct Cell Count (DCC) testing are consistent with established diagnostic thresholds for subclinical and clinical mastitis reported in the literature [5,8,36].

While SCC was employed as a diagnostic tool for detecting subclinical mastitis, it was not analyzed in relation to bacterial species or resistance profiles. Future studies may consider integrating SCC data with microbiological findings to explore potential predictive correlations.

Polymicrobial mastitis, observed in 22% of cases, may involve complex microbial interactions that go beyond mere co-isolation. Biofilm formation by one or more species can enhance antibiotic tolerance, while metabolic cooperation and antibiotic shielding may allow less-resistant species to persist during treatment. These mechanisms could contribute to clinical persistence and underscore the need for diagnostic and therapeutic approaches that consider interspecies interactions.

Our data show that *Streptococcus* spp. more frequently involved multi-quarter infections compared to *S. aureus* (Table 3). This may reflect a combination of factors including anatomical spread via contiguous ducts, higher environmental load, and the less robust immune evasion mechanisms of streptococci, which may allow easier dissemination across quarters [3]. In contrast, *S. aureus* often establishes persistent, localized infections with deep tissue involvement, facilitated by biofilm formation and immune evasion. These differences underscore the need for species-specific control measures tailored to pathogen ecology and transmission dynamics.

The lack of selective media or enrichment steps may have biased detection toward dominant or fast-growing species, particularly *S. aureus*, while potentially underestimating the presence of more fastidious or slower-growing pathogens such as certain streptococci or Gram-negative bacteria.

These cases likely reflect low pathogen loads, the presence of fastidious organisms not recovered by routine culture methods, or prior antimicrobial treatment that suppressed bacterial growth. Their inclusion is justified by standard field practice, where diagnosis often depends on indirect indicators such as CMT, SCC, and clinical examination, particularly in subclinical or previously treated cases.

A total of 10 milk samples (8.3%) were bacteriologically sterile, although the cows met the inclusion criteria for mastitis. Most of these cases were classified as subclinical, based on positive indirect tests (R-Mastitest and/or Mast-O-Test, CMT ≥ ++), elevated somatic cell counts, or mild clinical signs.

Culture-negative (“no growth”) mastitis is a well-recognized finding in both clinical and subclinical cases. Previous reports indicate that approximately 10–40% of milk samples from mastitic cows may yield no bacterial growth on routine culture, despite evidence of intramammary inflammation [42,43,44,45,46]. In our study, 8.33% of samples were culture-negative, yet SCC values remained elevated (358,000–1,420,000 cells/mL; mean 820,000 cells/mL), supporting an inflammatory process. Such outcomes may reflect low bacterial loads below detection limits, intermittent shedding, prior antimicrobial exposure, or the presence of fastidious organisms not recoverable under routine aerobic culture conditions, as well as pre-analytical factors related to sampling and handling [42,43,44,47].

The elevated SCC observed in culture-negative samples suggests that these cases represented true inflammatory intramammary processes despite the absence of detectable bacterial growth. Subclinical mastitis is characterized by the absence of visible clinical signs but is typically associated with elevated SCC, reflecting intramammary inflammation [16,20,47].

Culture-negative mastitis is a recognized phenomenon and may result from low bacterial load below the detection threshold, intermittent bacterial shedding, prior antimicrobial treatment suppressing viable organisms, or the presence of fastidious pathogens not recoverable under routine culture conditions. Additionally, inflammatory responses may persist after partial pathogen clearance, leading to sustained somatic cell elevation in the absence of viable bacteria.

The presence of positive indirect diagnostic tests (CMT and elevated SCC) in the absence of bacterial growth represents a recognized phenomenon known as culture-negative mastitis. In early stages of intramammary infection, the inflammatory response may precede detectable bacterial proliferation, resulting in elevated SCC despite low or transient pathogen loads. Furthermore, intermittent bacterial shedding, prior partial antimicrobial exposure, or the presence of fastidious organisms not recoverable under routine aerobic culture conditions may contribute to sterile culture results. Similar observations have been reported in previous studies, where 10–30% of mastitis samples yielded no bacterial growth despite clear inflammatory indicators. Therefore, elevated SCC in microbiologically negative samples should not be interpreted as absence of pathology, but rather as a limitation of conventional culture sensitivity [5,8,21,26,36].

The frequency of mastitis during late lactation (69%) observed in this study is atypical when compared to classical patterns, which report a peak incidence during early lactation. This discrepancy may be explained by local factors such as inadequate milking hygiene and delayed intervention protocols.

Several physiological and management-related factors contribute to the susceptibility of the mammary gland to intramammary infections during the dry period, particularly during the first two weeks after weaning, the so-called active involution phase [17,48,49]. First, although milking has ceased, the mammary gland continues to synthesize milk, reaching peak accumulation approximately 2–3 weeks after drying off. The resulting intramammary pressure can cause dilation of the teat canal and weakening of the teat sphincter, creating a pathway for ascending bacterial invasion. In the absence of regular milking, this ascending flora is no longer mechanically eliminated, and post-milking teat disinfection is typically discontinued, further exacerbating vulnerability to infection.

During the early dry period, the mammary gland undergoes active involution characterized by increased intramammary pressure, dilation of the teat canal, and altered immune cell functionality, which collectively enhance susceptibility to ascending bacterial invasion [23,36]. Phagocytic efficiency may be temporarily reduced during this phase, further predisposing the gland to infection. Although immunoglobulin and lactoferrin concentrations in the glandular secretions increase during involution and are believed to enhance immune defense, their protective effect may be insufficient under these altered physiological conditions. Importantly, while blanket dry cow therapy reduces the incidence of infections caused by *Staphylococcus* spp. and *Streptococcus* spp., it offers limited protection against coliform bacteria.

The VITEK^®^ 2 automated system used for bacterial identification and antimicrobial susceptibility testing has demonstrated high diagnostic performance in veterinary microbiology, with reported identification sensitivity ranging between 92 and 98% and specificity between 90 and 97%, depending on bacterial species [29,50,51,52]. Concordance with reference broth microdilution methods for antimicrobial susceptibility testing generally exceeds 90% in both Gram-positive and Gram-negative isolates. These characteristics support its reliability for routine mastitis pathogen characterization.

A similar set of vulnerabilities is observed during colostrogenesis the late gestation period during which colostrum synthesis begins. The progressive increase in glandular volume and pressure can lead to passive leakage of colostrum, coupled with the dilation of the teat duct. While citrate levels rise, lactoferrin levels drop, impairing bacteriostatic activity. Additionally, the phagocytic cells present at this stage are not fully competent in microbial defense, and despite high concentrations of immunoglobulins in colostrum, they do not effectively prevent the establishment of intramammary infections. Moreover, residual concentrations of antibiotics administered at dry-off may be insufficient to inhibit pathogen growth during this phase [30,53,54].

Metabolic disorders occurring during the transition period, particularly clinical and subclinical ketosis, have been associated with an increased risk of mastitis [55,56,57]. Negative energy balance and elevated circulating ketone bodies may impair neutrophil function and reduce immune competence, thereby increasing susceptibility to intramammary infections. Several studies have demonstrated that cows with subclinical ketosis exhibit a higher incidence of both clinical and subclinical mastitis compared to metabolically stable animals, highlighting the close interplay between metabolic status and udder health [55,56,57].

### Study Limitations

This study has certain limitations that must be acknowledged. First, its cross-sectional design, focused on five localities within Caraș-Severin County, limits the extrapolation of findings to other regions of Romania. Second, although VITEK^®^ 2 provided reliable phenotypic profiles, molecular characterization of resistance genes (e.g., *mecA*, *blaCTX-M*) was not performed, potentially underestimating certain resistance mechanisms. Third, environmental and management-related factors, which are known to influence mastitis risk and pathogen dynamics, were not systematically assessed, precluding more integrated interpretations. Addressing these gaps in future longitudinal studies would strengthen the evidence base and further align with reviewer recommendations regarding ecological and molecular dimensions of mastitis research.

This study has certain limitations due to its retrospective nature. The analysis relies on the accuracy and completeness of historical records, which may be subject to recording bias or misclassification. Additionally, observational design limits the ability to control confounding factors. Future prospective studies are recommended to validate these findings under controlled conditions.

## 4. Materials and Methods

This retrospective design reflects a cross-sectional snapshot of mastitis cases submitted for analysis, with no follow-up or repeated sampling. Therefore, associations observed between pathogen types and lactation stages should be interpreted descriptively, not causally. Further longitudinal studies are needed to confirm whether these patterns reflect temporal dynamics or underlying risk factors. This retrospective study is based on our data on the cytology of bacterial mastitis in cows. It was conducted from 3 October 2023 to 29 April 2024. Investigations included clinical examinations, laboratory tests, and calculation of epidemiological indices. The study did not include repeated tests or examinations on the same cows over time. Only complete records containing clinical signs, CMT results, treatment history, and bacteriological findings were included. Records with missing or ambiguous entries were excluded. Data validation was performed by comparing entries with veterinary logs and farm software (Uniform-Agri Milk Recording, Assen, The Netherlands, 5.0) to minimize inconsistencies.

Our research on the cytology of bacterial mastitis in cows was carried out during the period October 2023–April 2024.

To establish the lactocytogram for normal and pathological milk, the following objectives were fulfilled [46]:

- Individual observation sheets.
- Diagnostic tests for mastitis.
- Bacteriological examination for the collected milk samples.
- Cytological examinations for the samples taken under study.
- Centralizing the data.

The initial diagnosis of mastitis was based on clinical examination of the udder, including signs such as swelling, heat, pain, and abnormalities in the milk (e.g., clots or discoloration). The CMT (AHDB, Fontainebleau France) was used as a screening tool, and cows with a CMT score of at least ++ in one or more quarters were selected for further evaluation. In selected cases, milk samples were subjected to bacteriological culture to confirm infection.

Indirect detection of subclinical mastitis was performed using R-Mastitest and/or Mast-O-Test (Albert Kerbl GmbH, Buchbach, Germany). Both tests are based on the colorimetric detection of elevated somatic cell activity, reflecting the inflammatory status of the udder.

The tests use reagent cards that react with milk components, particularly enzymes such as n-acetyl-β-D-glucosaminidase and other somatic cell markers. The resulting color change is interpreted visually, using a scale provided by the manufacturer.

According to the kit instructions, test results were classified as follows:

- Negative: No color change or light yellow/green.
- Suspect: Intermediate color (light purple, pale orange).
- Positive: Strong color change (blue, dark purple, red), indicating high cell count/inflammation.

These tests were used in combination with clinical signs and/or CMT to classify cows as having clinical or subclinical mastitis. Only animals with a positive result on at least one indirect test, or clear clinical signs, were included.

**Figure 2:**
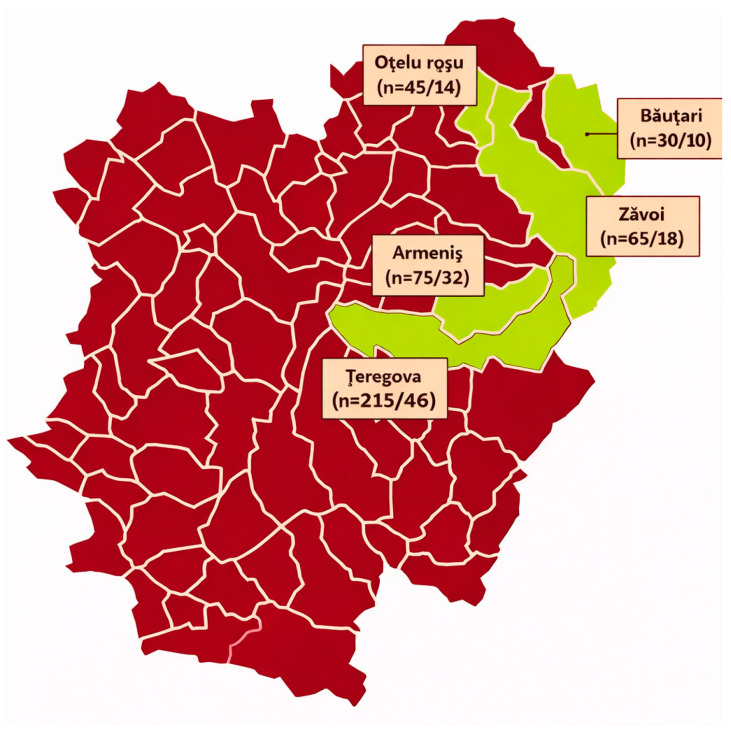
Locations from where milk samples from cows with mastitis were gathered.

Of a total of 420 cows investigated for mastitis, 120 cows were classified as mastitis providing from five localities from Northeast Caraș Severin County, (45.1140° N, 22.0741° E), Banat region, Romania: Oțelu Roșu (*n* = 45/14), Zăvoi (*n* = 65/18) Băuțari (*n* = 30/10), Teregova (*n* = 215/46), Armeniș (*n* = 75/32) (Figure 2).

### 4.1. Inclusion/Exclusion Criteria

The 120 milk samples from mastitis classified cows, were processed and statistically interpreted. The rest of the samples were ignored, either due to incomplete bacteriological examination, which identified only the bacterial genera or uncertainty, or due to faltering cytological examination, mainly due to the very low somatic cells in the milk smears.

Lactating cows of the Holstein-Friesian and Romanian Spotted breeds were included in the study. All animals were in early lactation (≤100 days in milk), clinically healthy aside from signs of mastitis, and had not received any antibiotic treatment within the previous 30 days. Cows with systemic illness, metabolic disorders, or other concurrent conditions were excluded from the study.

SCC were determined on-farm using a DeLaval Cell Counter (DCC; DeLaval International AB, Tumba, Sweden), based on fluorescence-based cell counting technology. Results were expressed as cells/mL and were used as a complementary diagnostic parameter for differentiating subclinical and clinical mastitis cases. SCC values were not subjected to statistical analysis or correlated with antimicrobial resistance profiles in the present study.

### 4.2. The Individual Observation Sheets

To have a record of the milk samples collected, individual observation sheets were prepared for the examined cows. In these sheets, anamnestic data necessary later to obtain an overview of the epidemiological situation regarding mastitis in the cow farms studied were entered. The individual observation sheets designed were completed, each time, and included the data presented in Figure S1—Supplementary File.

The study was conducted on bacterial isolates obtained from clinically confirmed cases diagnosed within the target population included in the present investigation. Biological samples were collected aseptically from affected animals, depending on the clinical localization of the infection, using sterile swabs or collection containers in accordance with standard veterinary microbiology protocols. Immediately after sampling, the specimens were transported under refrigerated conditions to the microbiology laboratory and processed within 24 h to preserve bacterial viability and reduce the risk of contamination or overgrowth of secondary flora.

Bacterial cultures were performed using standard blood agar and MacConkey agar. No selective or differential media were used for the enhanced recovery of *Streptococcus* spp., and no enrichment protocols were applied for Gram-negative bacteria. This may have favored the isolation of fast-growing and abundant organisms such as *S. aureus*.

Primary isolation was performed on appropriate culture media selected according to the presumptive clinical diagnosis, including non-selective and selective agar plates suitable for both Gram-positive and Gram-negative bacteria. The inoculated media were incubated aerobically at 37 °C and monitored at 24 and 48 h for bacterial growth. Macroscopic examination of colonies included the assessment of morphology, pigmentation, hemolysis patterns, and consistency. Suspected colonies were subcultured to obtain pure isolates for subsequent identification and antimicrobial susceptibility testing.

Bacterial identification was performed using the VITEK^®^ 2 Compact automated system (bioMérieux, Marcy-l’Étoile, France), based on biochemical profiling. Colonies grown on blood agar or MacConkey agar were selected and suspended in 0.45% sterile saline to a turbidity equivalent to 0.5 McFarland standard, as measured by a densitometer. The standardized suspensions were loaded into ID-GP or ID-GN cards, depending on Gram stain results.

Cards were automatically incubated and read by the VITEK^®^ 2 system using the AST-GP and AST-GN database (software version 9.01). Identification was considered reliable when the confidence level provided by the system was ≥95%. For isolates with ambiguous profiles or borderline results, confirmatory testing using conventional biochemical assays was performed.

Antimicrobial susceptibility was interpreted using veterinary-specific CLSI breakpoints (CLSI VET01 and VET08), appropriate for bovine mastitis pathogens [29]. Human clinical breakpoints were not used in this analysis. In addition, resistance profiles were interpreted in the context of potential multidrug resistance, defined as resistance to three or more antimicrobial classes. Results generated by the automated system were reviewed for biological plausibility, and internal quality control procedures were applied throughout the analysis process using reference strains to verify system performance.

Antimicrobials were categorized according to the World Health Organization (WHO) list of Critically Important Antimicrobials for Human Medicine [58], which prioritizes classes such as third-generation cephalosporins, fluoroquinolones, and macrolides due to their essential role in treating serious human infections. This classification was used to assess the broader One Health implications of resistance patterns observed in bovine mastitis pathogens.

All laboratory procedures were performed in compliance with institutional biosafety regulations. The study design involved only diagnostic samples obtained as part of routine clinical investigations, with no experimental infection or animal intervention, and therefore did not require formal ethical approval according to national guidelines.

### 4.3. Statistical Data Analysis

Statistical analysis was performed using Microsoft Excel 2016 and GraphPad Prism version 9 (GraphPad Software Inc., San Diego, CA, USA).

The statistical unit of analysis was the individual milk sample, corresponding to a single under quarter. Each sample was treated independently, and when multiple isolates were recovered from a sample, each was considered as a distinct observation. This quarter-level approach aligns with the sample submission protocol and underpins all statistical comparisons, including Fisher’s exact test and the calculation of confidence intervals.

## 5. Conclusions

This retrospective study provides a current view of the bacteriological profile and antimicrobial resistance patterns associated with bovine mastitis in the Banat region of Romania.

*S. aureus* and *S. agalactiae* were identified as the predominant mastitis pathogens, confirming their ongoing epidemiological relevance. Notably, beta-lactam resistance was widespread among both Gram-positive and Gram-negative isolates, with high levels of resistance to penicillin and ampicillin. The presence of MDR strains further raises concern regarding therapeutic efficacy and highlights the need for improved antimicrobial stewardship in veterinary practice.

These findings emphasize the importance of routine bacteriological testing and local resistance monitoring as essential components of mastitis control programs. Evidence-based antimicrobial use, tailored to regional resistance profiles, is strongly recommended to preserve treatment effectiveness and reduce the spread of resistant strains.

## Figures and Tables

**Table 1:** Bacteriological Profile of Mastitis Cases: Pathogen Distribution and Culture Results.

Bacterial Species	Number of Isolates (*n*)	Percentage (%)	Gram Type
*S. aureus*	32	26.6%	Gram-positive
*S. agalactiae*	24	20%	Gram-positive
*E. coli*	18	15%	Gram-negative
*S. dysgalactiae*	9	7.5%	Gram-positive
*S. uberis*	8	6.6%	Gram-positive
*S. pyogenes*	4	3.3%	Gram-positive
Polymicrobial infection	5	4.1%	—
Sterile samples	10	8.3%	—
Total	120	100%	

**Table 2:** MDR Profile of Bacterial Isolates Recovered from Bovine Mastitis Cases.

Species	No. of Isolates	MDR Isolates (≥3 Classes)	% MDR
*S. aureus*	40	15	37.5%
*S. agalactiae*	27	6	22.2%
*S. dysgalactiae*	3	1	33.3%
*S. uberis*	6	2	33.3%
*S. pyogenes*	3	1	33.3%
*C. bovis*	2	0	0%
*E. coli*	1	1	100%
*K. pneumoniae*	1	1	100%
*M.morganii*	1	1	100%

**Table 3:** Frequency of mastitis and occurrence of new infections, depending on the lactation phase in cattle.

The Etiological Agent	Lactation Phase/Days					
	I (30–60)	II (60–245)	III (245–305)	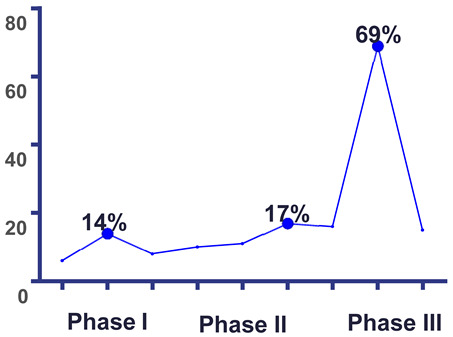		
*S. aureus*	2	9	28			
*Streptococcus * spp *.*	10	5	25			
*C. bovis*	-	-	2			
*E. coli*	-	1	-			
*K. pneumoniae*	-	-	1			
*M. morganii*	-	-	1			
Polymicrobial infection	-	4	22			
Sterile samples	5	1	4			
Total cases	17	20	83	I (30–60)	II (60–245)	III (245–305)
Frequency (%)	14	17	69			

## Data Availability

The data presented in this study are included in the article. Further inquiries can be directed to the corresponding author.

## Supplementary Materials

The following supporting information can be downloaded at: https://www.mdpi.com/article/10.3390/antibiotics15020198/s1, Figure S1: Clinical observation sheet; Figure S2: Distribution of Bacterial Species Isolated from Bovine Mastitis Cases; Figure S3: Distribution of Culture Outcomes in Bovine Mastitis Samples; Figure S4: Forest Plot of MDR Proportions with 95% CI Across Bacterial Species; Table S1: Antimicrobial susceptibility profile and estimated MIC values of bacterial isolates determined with Vitek 2 (AST-GP and AST-GN); Table S2: Minimum Inhibitory Concentrations (MICs) and CLSI Interpretations for Gram-Negative Bacterial Isolates Recovered from Bovine Mastitis Cases (VITEK AST-GN Panel); Table S3: Species-Level Distribution of Bacterial Isolates Classified as Susceptible or MDR (≥3 Antibiotic Classes); Table S4: Proportion of MDR Isolates by Bacterial Species, 95% Confidence Intervals, and Statistical Comparisons (*p*-values); Table S5: Antibiotic Classes Tested and MDR Status of Bacterial Isolates Recovered from Bovine Mastitis Cases.

## Abbreviations

The following abbreviations are used in this manuscript:

AMR	Antimicrobial Resistance
AST-GP	Antimicrobial Susceptibility Testing—Gram Positive (VITEK card)
AST-GN	Antimicrobial Susceptibility Testing—Gram Negative (VITEK card)
CLSI	Clinical and Laboratory Standards Institute
MDR	Multidrug-Resistant
MIC	Minimum Inhibitory Concentration
SCC	Somatic Cell Count
