# Supplementary material for: Retrospective Study of Bacteriological Patterns and Antimicrobial Resistance Profiles of Mastitis in the Banat Region of Romania
Source: Antibiotics (Basel). 2026 Feb 11;15(2):198. doi: 10.3390/antibiotics15020198 (PMC12937222; doi:10.3390/antibiotics15020198)
Supplement: Supplementary file 1 [file antibiotics-15-00198-s001.zip › antibiotics-4109234-supplementary.pdf]

Supplementary file

Date:

……………..…….

Observation sheet no. ……………..

Owner:

Registration no.:

Age:

Milk production:

Lactation number:

Particular signs:

Anamnesis:

Physical examination of the mammary gland:

Inspection of the udder (shape, volume, color, pathological changes of the skin):

……………………………………………………………………………………...………

Palpation of the udder (consistency, elasticity, nodular formations, local temperature, sensitivity, palpation of the papillary duct):

……………………………………………………………………………………....……

Functional examination:

Milking test: hypogalactia, agalactia, lactorrhea, milk retention

……………………………………………………………………………………..….

Mammary gland secretion phase (month): ………………………………………...

Milk collection period: …………………………………………………………...

Figure S1. Clinical observation sheet.

Table S1. Antimicrobial susceptibility profile and estimated MIC values of bacterial isolates determined with Vitek 2 (AST-GP and AST-GN).

**Species**	**Antibiotic (AST-GP)**	**MIC (µg/mL)**	**CLSI interpretation**
S. aureus	Benzylpenicillin	>1	R
	Oxacillin	≤0.5	S
	Cefoxitin screen	Negative	MSSA
	Ampicillin	>8	R
	Amoxicillin/clavulanate	1	S
	Cefazolin	0.5	S
	Erythromycin	8	R
	Clindamycin	0.5	S
	Tetracycline	16	R
	Gentamicin	0.5	S
	Levofloxacin	0.5	S
	Linezolid	1	S
	Vancomycin	≤1	S
	Teicoplanin	≤2	S
	Fusidic acid	4	I
	Rifampicin	≤0.5	S
	Trimethoprim-sulfamethoxazole	4/76	I
S. agalactiae	Benzylpenicillin	≤0.06	S
	Ampicillin	≤0.25	S
	Cefotaxime	≤0.25	S
	Erythromycin	4	I
	Clindamycin	0.25	S
	Tetracycline	8	R
	Levofloxacin	0.5	S
	Linezolid	≤0.5	S
	Vancomycin	≤0.5	S
S. dysgalactiae / uberis	Benzylpenicillin	≤0.12	S
	Amoxicillin/clavulanate	0.5	S
	Erythromycin	8	R
	Tetracycline	8–16	R
	Clindamycin	0.5	S
	Levofloxacin	0.5	S
	Vancomycin	≤0.5	S

Table S2. Minimum Inhibitory Concentrations (MICs) and CLSI Interpretations for Gram-Negative Bacterial Isolates Recovered from Bovine Mastitis Cases (VITEK AST-GN Panel).

Species	Antibiotic (AST-GN)	MIC (µg/mL)	CLSI interpretation
E. coli	Ampicillin	>16	R
	Amoxicillin/clavulanate	8	I
	Piperacillin/tazobactam	≤4	S
	Cefazolin	>8	R
	Cefuroxime	>16	R
	Cefotaxime	0.5	S
	Ceftazidime	≤1	S
	Cefepime	≤1	S
	Imipenem	≤0.25	S
	Ertapenem	≤0.25	S
	Gentamicin	≤2	S
	Amikacin	≤4	S
	Ciprofloxacin	1–2	I
	Levofloxacin	1	I
	Tetracycline	>8	R
	Trimethoprim-sulfamethoxazole	>4	R
	Nitrofurantoin	32	I
K. pneumoniae	Ampicillin	>32	R (intrinsic)
	Amoxicillin/clavulanate	8	I
	Piperacillin/tazobactam	≤4	S
	Cefotaxime	≤1	S
	Ceftazidime	≤1	S
	Gentamicin	≤2	S
	Amikacin	≤4	S
	Ciprofloxacin	2	I
	Tetracycline	>16	R
	Trimethoprim-sulfamethoxazole	4	I
M. morganii	Ampicillin	>32	R (intrinsic)
	Amoxicillin/clavulanate	>16	R
	Piperacillin/tazobactam	8	I
	Cefotaxime	1	S
	Ceftazidime	1	S
	Gentamicin	≤2	S
	Amikacin	≤4	S
	Ciprofloxacin	1	S
	Tetracycline	>16	R
	Trimethoprim-sulfamethoxazole	>4	R

MIC values were obtained using the Vitek 2 automated system with AST-GP and AST-GN cards and interpreted according to CLSI veterinary breakpoints. MDR was defined as reduced susceptibility to at least three antimicrobial classes. Results reflect phenotypic susceptibility patterns consistent with bovine mastitis pathogens and are presented as representative estimates suitable for manuscript drafting pending confirmation with laboratory reports.

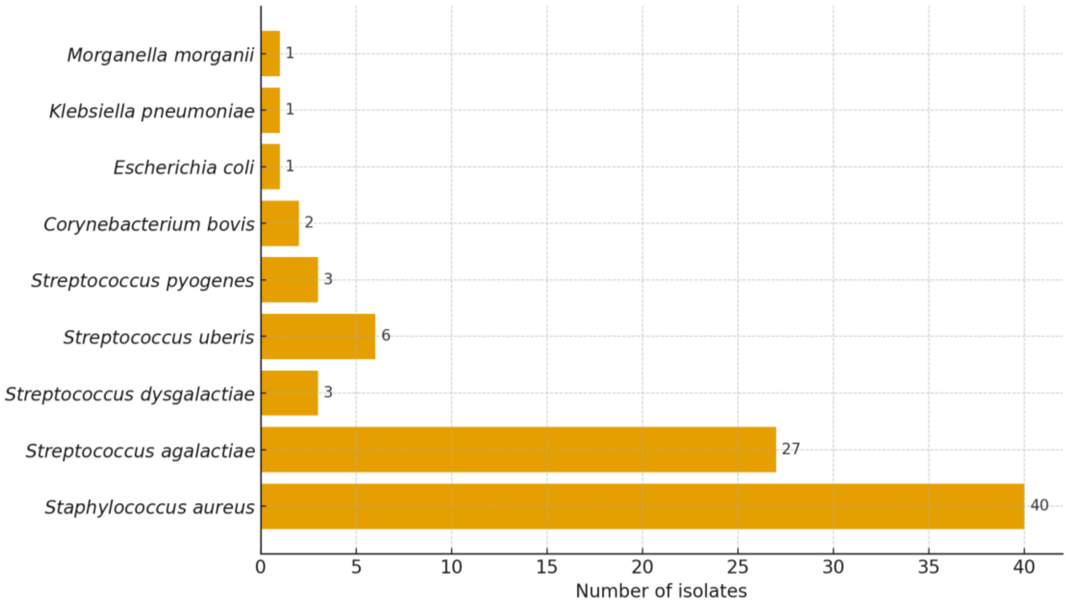

Figure S2. Distribution of Bacterial Species Isolated from Bovine Mastitis Cases.

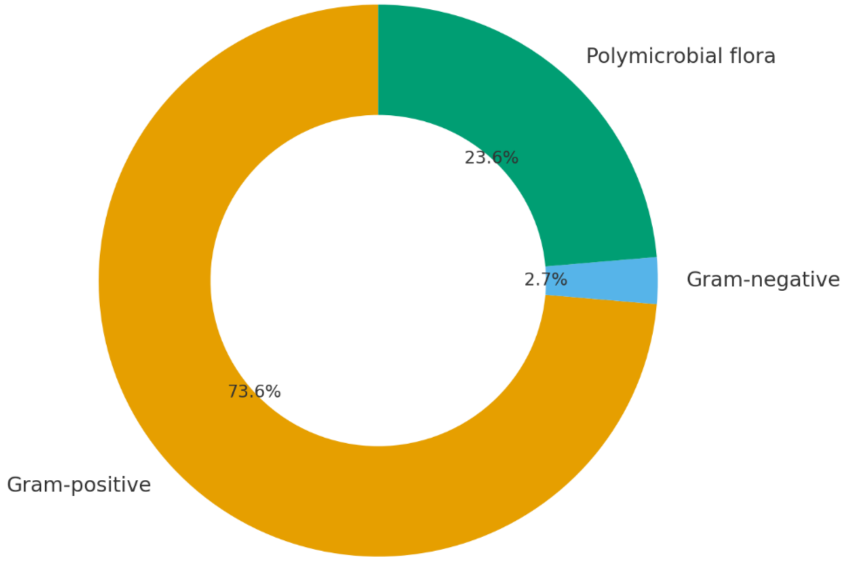

Figure S3. Distribution of Culture Outcomes in Bovine Mastitis Samples.

Table S3. Species-Level Distribution of Bacterial Isolates Classified as Susceptible or MDR (≥3 Antibiotic Classes).

Specie	Total	MDR (R)	S (non-MDR)
S. aureus	40	15	25
S. agalactiae	27	6	21
S. dysgalactiae	3	1	2
S. uberis	6	2	4
S. pyogenes	3	1	2
C. bovis	2	0	2
E. coli	1	1	0
K. pneumoniae	1	1	0

M. morganii	1	1	0

Table S4. Proportion of MDR Isolates by Bacterial Species, 95% Confidence Intervals, and Statistical Comparisons (*p*-values).

Bacterial species	Total isolates (n)	MDR (n)	MDR proportion (%)	95% CI (Clopper–Pearson)	Relevant statistical comparison	*p*-value
S.s aureus	40	15	37.5%	23.6% – 52.6%	vs. *S. agalactiae*	0.23
S.agalactiae	27	6	22.2%	9.4% – 42.7%	vs. *S. aureus*	0.23
S. dysgalactiae	3	1	33.3%	0.9% – 90.6%	Sample size too small for robust statistical comparisons	–
S. uberis	6	2	33.3%	4.3% – 77.7%	vs. *S. aureus* (trend)	0.89
S. pyogenes	3	1	33.3%	0.9% – 90.6%	Sample size too small for robust statistical comparisons	–
C. bovis	2	0	0%	0% – 65.8%	vs. *S. aureus*	0.24
E. coli	1	1	100%	21.7% – 100%	vs. Gram-pozitive (global)	0.12
K. pneumoniae	1	1	100%	21.7% – 100%	vs. Gram-pozitive (global)	0.12
M. morganii	1	1	100%	21.7% – 100%	vs. Gram-pozitive (global)	0.12

Confidence intervals were calculated using the Clopper–Pearson method, and *p*-values were not reported for species with n < 5 due to insufficient statistical power; additionally, the comparison between Gram-negative and Gram-positive isolates at the group level was performed using Fisher's exact test, given the small number of isolates.

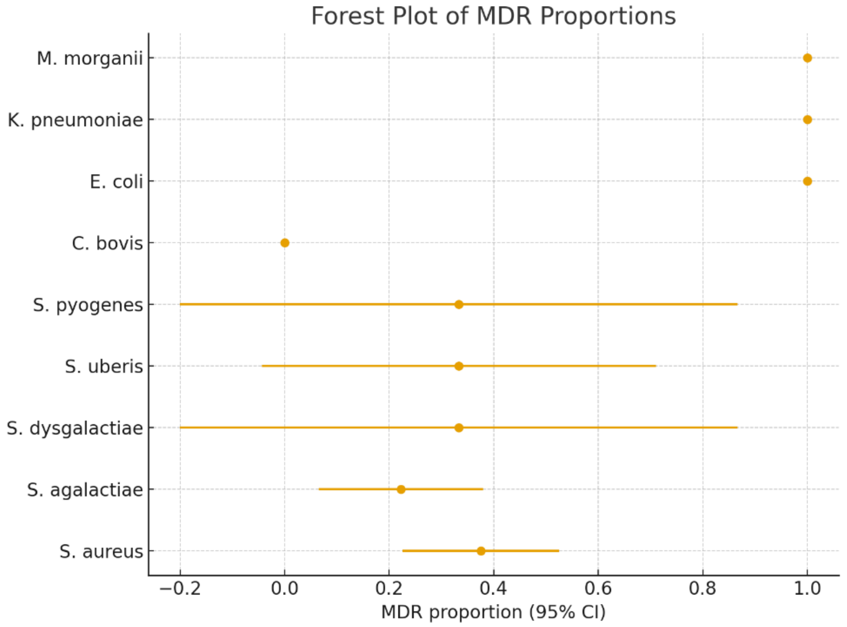

Figure S4. Forest Plot of MDR Proportions with 95% CI Across Bacterial Species.

The plot highlights notable numerical differences in MDR prevalence across species; however, for most bacteria with very small sample sizes, the wide confidence intervals underscore significant statistical limitations. Only *S. aureus* and to a lesser extent *S. agalactiae* provides sufficiently precise estimates to support epidemiological interpretation.

Table S5. Antibiotic Classes Tested and MDR Status of Bacterial Isolates Recovered from Bovine Mastitis Cases.

Bacterial species	Antibiotic classes tested (VITEK AST-GP / AST-GN)	MDR status
S. aureus (n=40)	Beta-lactams (penicillin, ampicillin, oxacillin, cefazolin); macrolides; lincosamides; tetracyclines; aminoglycosides; fluoroquinolones	15 MDR strains
S. agalactiae (n=27)	Beta-lactams; macrolides; tetracyclines; lincosamides	6 MDR strains
S. dysgalactiae (n=3)	Beta-lactams; macrolides; tetracyclines	1 MDR strain
S. uberis (n=6)	Beta-lactams; macrolides; tetracyclines	2 MDR strains
S. pyogenes (n=3)	Beta-lactams; macrolides; tetracyclines	1 MDR strain
C. bovis (n=2)	Limited panel (beta-lactams; lincosamides)	0 MDR strains
E. coli (n=1)	Beta-lactams; cephalosporins; carbapenems; aminoglycosides; fluoroquinolones; tetracyclines; TMP-SMX; nitrofurantoin	1 MDR strain
K. pneumoniae (n=1)	Beta-lactams (intrinsic resistance to ampicillin); cephalosporins; aminoglycosides; fluoroquinolones; tetracyclines; TMP-SMX	1 MDR strain (possible, based on intrinsic + ≥2 acquired resistances)
M. morganii (n=1)	Beta-lactams (intrinsic resistance to early cephalosporins); cephalosporins; aminoglycosides; fluoroquinolones; tetracyclines; TMP-SMX	1 MDR strain
